# 

**Published:** 1906-04

**Authors:** 


### THE MEDICAL ASSOCIATION OF GEORGIA.

THE next meeting of the Medical Association of Georgia will be held in Augusta. The following is the preliminary program:

Syphilis Among Negroes and How to Better their Condition. D. D. Quillian, M.D., Athens.

Recent Studies in Rabies. Jas. N. Brawner, M.D., Atlanta.

The Treatment of Neuritis and After Congestive Conditions, with the Static Modalities. R. P. Izler, M.D., Waycross.

A New and Original Simplification of the Present Methods of Infant Feeding. Chas. E. Boynton, M.D., Atlanta.

Dementia Præcox. J. W. Mobley, M.D., Milledgeville.

The Significance of Fever. W. H. Doughty, Jr., M.D., Augusta.

The Necessity for Early Diagnosis of Pulmonary Tuberculosis. T. E. Oertel, M.D., Augusta.

Enteroptosis. L. Amster, M.D., Atlanta.

A New Treatment of Typhoid Fever; 55 Cases, No Deaths. Chas. Boyd, M.D., Bowen's Mill.

Mastoiditis and the Indications for Performing the Mastoid Operation. Frank M. Cunningham, M.D., Macon.

The Significance of Albumin and Casts in the Urine. W. A. Mulherin, M.D., Augusta.

A Case of Popliteal Aneurysm Occurring in my Practice: Cure. J. C. Groves, M.D., Lincolnton.

The Treatment of Broncho-Pneumonia. S. A. Visanska, M.D., Atlanta.

Anterior Polio-Myelitis. J. Cheston King, M.D., Atlanta.

The General Practitioner as a Factor in Surgery. R. R. Kime, M.D., Atlanta.

Is There a Continued Fever Other than Malarial or Typhoid? J. W. Palmer, M.D., Ailey.

Circumcision. J. H. Crawford, M.D., Martin.

Puerperal Eclampsia. Pryor W. Fitts, M.D., Greenville.

Hernia; My Observations and Results. W. B. Crawford, M. D., Savannah.

Therapeutic Suggestion in the Practice of Medicine. G. T. Kesner, M.D., Decatur.

Medical Ethics. M. A. Clark, M.D., Macon.

Paramyoclonus Multiplex, with Report of a Case. E. Bates Block, M.D., Atlanta.

Antipyresis and its Feasibility. R. H. Thigpen, M.D., Augusta.

The Antiseptic and Eliminative Treatment of Typhoid Fever. J. B. Warnell, M.D., Register.

Light and Vibration, Mechanical and Chemical, as Therapeutic Agents. J. R. Sewell, M.D., Carrollton.

The Diagnosis and Treatment of Gall Stones. Geo. R. White, M.D,. Savannah.

Hyperemesis Gravidarum. Ralston Lattimore, M.D., Savannah.

Differential Diagnosis of Gall Stones. J. T. Rogers, M.D., Savannah.

Acidosis. W. R. Houston, M.D., Augusta.

Treatment of Constipation. Theodore Toepel, M.D., Atlanta.

Post-Operative Phlebitis. J. R. Garner, M.D., Atlanta.

Intussusception, with Special Reference to Diagnosis. Marion McH. Hull, M.D., Atlanta.

Criminal Abortion. E. Morgan, M.D., Arlington.

The Antrum and its Treatment, with Special Reference to Accessory Complications. Arthur G. Hobbs, M.D., Atlanta.

The Cystoscope and its Uses. E. G. Ballenger, M.D., Atlanta.

Tuberculosis. J. W. Hurt, M.D., Atlanta.

The Giant Magnet as an Eye Preserver. Ross P. Cox, M.D., Rome.

The Influence of Respiration upon the Development of the Chest Deformity in Scoliosis, and its Relation to the Application of the Plaster Jacket for the Correction of the Deformity. Michael Hoke, M.D., Atlanta.

Some Remarks on Chronic Conditions. H. McHatton, M.D., Macon.

An Unusual Complication Following Typhoid Fever; Report of Case. J. E. Sommerfield, M.D., Atlanta.

Para-Typhoid, a Type of Continued Fever, its Clinical Distinctions. A. P. Taylor, M.D., Thomasville.

The Radical Cure of Middle Ear Suppuration. Dunbar Roy, M.D., Atlanta.

Antipyretics in Febrile Diseases. Jas. B. Baird, M.D., Atlanta.

Lesions Predisposing to Cancer. M. B. Hutchins, M.D., Atlanta.

Gastric Ulcer, its Diagnosis and Treatment. Wm. H. Moss, M.D., Atlanta.

Three Months' Colic in Babies. W. B. Parks, M.D., Atlanta.

Gonorrhea. W. C. DeLamar, M.D., Mountain Hill.

New Medical Era. J. G. Edie, M.D., Nashville.

Pneumonia, Carbonate of Ammonia in Same. H. L. McCrary, M.D., Royston.

Appendicitis. F. W. McRae, M.D., Atlanta.

Clinical Consideration of Tumors; Report of Cases. W. F. Westmoreland, M.D., Atlanta.

A Case Extraordinary of Laceration of Perineum with Loss of the Base of Bladder. Jno. G. Earnest, M.D., Atlanta.

Report of Five Cases of Fracture of Femur, with Remarks on Treatment and Exhibition of Extension and Counterextension Apparatus. J. H. Downey, M.D., Gainesville.

Report of a case of Addison's Disease. Wm. Clifton Lyle, M. D., Augusta.

Vaginal Sarcoma in an Infant Seventeen Months of Age. O. L. Holmes, M.D., Covington.

The Prognosis in Chronic Nephritis. Claude A. Smith, M.D., Atlanta.

Report of Emergency Cases at Grady Hospital. L. T. Pattillo, M.D., Atlanta.

Diagnosis and Treatment of Pulmonary Tuberculosis. H. F. Harris, M.D., Atlanta.

The Anatomy and Development of the Peritoneum and Abdominal Viscera. W. B. Armstrong, M.D., Atlanta.

A Simple Method of Straining Spirochætæ Pallida. C. R. Andrews, M.D., Atlanta.

Dysmenorrhea in Young Women. Willis Jones, M.D., Atlanta.

Radium. F. G. Hodgson, M.D., Atlanta.

---

AN International Exposition of Hygiene is to open at Milan in May and to continue through the summer. To celebrate the occasion the King of Italy has offered a prize of $1,000 for the best well-tested arrangement for supplying cities with pure milk. A second prize of $2,000 is also offered for the best type of dwelling house for the masses adapted to the climate of Southern Italy.—*Exchange.*

